# Competition in the *Phaseolus vulgaris*-*Rhizobium* symbiosis and the role of resident soil rhizobia in determining the outcomes of inoculation

**DOI:** 10.1007/s11104-023-05903-0

**Published:** 2023-02-03

**Authors:** George M. Mwenda, Yvette J. Hill, Graham W. O’Hara, Wayne G. Reeve, John G. Howieson, Jason J. Terpolilli

**Affiliations:** 1 Legume Rhizobium Sciences, Food Futures Institute, Murdoch University, 90 South Street, Murdoch, WA 6150 Australia; 2 Present Address: Department of Primary Industries and Regional Development, 75 York Road, Northam, WA 6401 Australia; 3 Food Futures Institute, Murdoch University, 90 South Street, Murdoch, WA 6150 Australia

**Keywords:** Rhizobia, Legume inoculation, Competition, N_2_ fixation, *celB*/*gusA* dual marker system

## Abstract

**Background and Aims:**

Inoculation of legumes with effective N_2_-fixing rhizobia is a common practice to improve farming profitability and sustainability. To succeed, inoculant rhizobia must overcome competition for nodulation by resident soil rhizobia that fix N_2_ ineffectively. In Kenya, where *Phaseolus vulgaris* (common bean) is inoculated with highly effective *Rhizobium tropici* CIAT899 from Colombia, response to inoculation is low, possibly due to competition from ineffective resident soil rhizobia. Here, we evaluate the competitiveness of CIAT899 against diverse rhizobia isolated from cultivated Kenyan *P. vulgaris*.

**Methods:**

The ability of 28 Kenyan *P. vulgaris* strains to nodulate this host when co-inoculated with CIAT899 was assessed. Rhizosphere competence of a subset of strains and the ability of seed inoculated CIAT899 to nodulate *P. vulgaris* when sown into soil with pre-existing populations of rhizobia was analyzed.

**Results:**

Competitiveness varied widely, with only 27% of the test strains more competitive than CIAT899 at nodulating *P. vulgaris*. While competitiveness did not correlate with symbiotic effectiveness, five strains were competitive against CIAT899 and symbiotically effective. In contrast, rhizosphere competence strongly correlated with competitiveness. Soil rhizobia had a position-dependent numerical advantage, outcompeting seed-inoculated CIAT899 for nodulation of *P. vulgaris,* unless the resident strain was poorly competitive.

**Conclusion:**

Suboptimally effective rhizobia can outcompete CIAT899 for nodulation of *P. vulgaris*. If these strains are widespread in Kenyan soils, they may largely explain the poor response to inoculation. The five competitive and effective strains characterized here are candidates for inoculant development and may prove better adapted to Kenyan conditions than CIAT899.

## Introduction

Legumes are a versatile group of plants that provide food for human consumption, pastures for grazing, and act as disease break crops in farming systems in many parts of the world (Angus et al. 2015; Broughton et al. 2003; Howieson et al. 2000). Legumes also boost agricultural productivity by supplying nitrogen to soils through a symbiotic relationship formed between the plant and a group of soil bacteria called rhizobia. The symbiosis is established when rhizobia infect legume roots, resulting in the formation of root nodules. Inside legume root nodules, rhizobia fix atmospheric N_2_ into NH_3_, which is secreted to the host plant and assimilated. Some of this fixed N_2_ makes its way into the soil in legume residues and can therefore benefit subsequent crops (Brophy and Heichel 1989; Maluk et al. 2022; Peoples et al. 2009). By harnessing symbiotic N_2_ fixation, farmers can reduce their reliance on synthetic nitrogen fertilizers, lower production costs, and contribute to global efforts to reduce carbon emissions.

To maximize the benefits of symbiotic N_2_ fixation in agriculture, legumes are often inoculated with highly effective strains of rhizobia that fix large amounts of N_2_ (Herridge et al. 2008; Howieson et al. 2000; Vanlauwe et al. 2019). However, rhizobia are common members of soil microbial communities and as such there can be great variation in the numerical abundance and genotypic composition of these soil communities (Brockwell et al. 1995; Kumar et al. 2015). When more than one genotype of rhizobia is capable of nodulating the target legume within the rhizosphere, competition for nodulation of the host plant may arise between the inoculant strain and these resident strains (Yates et al. 2011). When the inoculant strain is displaced in legume nodules by a more competitive resident strain that fixes substantially less N_2_ than the inoculant, then the full benefit of symbiotic N_2_ fixation cannot be realized, resulting in a diminished impact on farming systems.

In cases where resident soil rhizobia outcompete the inoculant strain for nodulation of the host, resident soil rhizobia are considered to create a “barrier” to inoculation response. Several studies have investigated overcoming this barrier. Weaver and Frederick (1974) working with soybean reported that > 10^3^ rhizobia were required on the seed relative to the soil population, to achieve a response to inoculation. Other investigators subsequently showed that soil with populations as low as 10^2^ rhizobia.g soil^−1^ (Singleton and Tavares 1986; Vargas et al. 2000) or even 50 rhizobia.g soil^−1^ (Thies et al. 1991a) were sufficient to provide a barrier to the inoculation response. In contrast, inoculation responses were achieved for *Phaseolus vulgaris* when inoculant was delivered into soils with 10^3^ rhizobia.g soil^−1^ (Hungria et al. 2003; Vlassak et al. 1996) and as many as 10^4^ and 10^5^ rhizobia.g soil^−1^ (Hungria et al. 2000). Although the reason why an inoculation response can be inhibited by such vastly different numbers of resident soil rhizobia is not clearly understood, competitiveness is a genetic trait (Mendoza-Suarez et al. 2021) which is known to vary widely across individual strains (Aguilar et al. 2022). Thus, while the number of resident rhizobia is a key consideration in nodulation outcomes following inoculation, the competitive ability of resident strains in nodulating the target legume is also an important consideration.

*Phaseolus vulgaris* (common bean) is a grain legume that is widely grown as a source of dietary protein. Originating from two centers of domestication in Mesoamerica and the Andes (Schmutz et al. 2014), *P. vulgaris* is now cultivated in many areas of the tropics and sub-tropics. *P. vulgaris* is considered a promiscuous legume, since it is nodulated by a broad range of rhizobial species and while most of these are in the genus *Rhizobium*, species of *Sinorhizobium* and *Bradyrhizobium* as well as *Paraburkholderia* and *Cupriavidis* have also been reported to nodulate this host (da Silva et al. 2012; Dall’Agnol et al. 2016; Martínez-Romero 2003). In Kenya, where *P. vulgaris* has been cultivated for 400–500 years, grain yields remain low due to a range of issues such as low yielding cultivars, disease pressure, drought and low soil fertility, including low soil nitrogen levels (Katungi et al. 2009; Margaret et al. 2014). Although Kenyan soils contain native rhizobia capable of nodulating *P. vulgaris*, a substantial proportion of these rhizobia appear to be suboptimally effective at fixing N_2_ (Anyango et al. 1995; Kawaka et al. 2014). Therefore, *P. vulgaris* is sometimes inoculated with *Rhizobium tropici* CIAT899, a highly effective N_2_-fixing strain isolated from *P. vulgaris* in Colombia (Martínez-Romero et al. 1991). However, the yield of inoculated plants remains low, possibly due to poor adaptability of the inoculant strain to local environmental conditions and the presence of reasonably high (i.e. 10^4^ cells.g soil^−1^) populations of resident rhizobia in soils that presumably compete for nodulation of *P. vulgaris* (Anyango et al. 1998; Musandu and Ogendo 2001). It is therefore possible that the lack of an inoculation response is due to a high population of competitive and ineffective N_2_ fixing rhizobia in Kenyan soil, creating a barrier to the successful nodulation of the host plant by the commercial inoculant strain.

Previously, we characterized the genotypic diversity of 36 rhizobia strains isolated from cultivated *P. vulgaris* sampled from 16 sites across five different counties in Kenya (Mwenda et al. 2018). We showed that these strains represented at least five different known *Rhizobium* spp. and at least one novel *Rhizobium* spp. Importantly, a relatively high proportion of these strains (22 of 36 or 61%) were effective at fixing N_2_, with 10 strains fixing N_2_ at rates equivalent to that of CIAT899. Thus, diverse and effective indigenous rhizobia nodulate cultivated *P. vulgaris* in Kenya. However, the infrequency with which inoculation with CIAT899 yields a positive response suggests the presence of competitive yet poorly effective strains in the soil. Here, we adapted a *celB* and *gusA* dual marker system (Sanchez-Canizares and Palacios 2013) to investigate competitiveness of this diverse range of *P. vulgaris* nodulating rhizobia from Kenya, in controlled glasshouse co-inoculation experiments with CIAT899.

## Materials and methods

### Bacterial strains, plasmids and media

Strains and plasmids used in the study are listed in Table 1. *Escherichia coli* strains were routinely cultured in Lysogeny Broth (LB) media at 37˚C (Bertani 1951) and rhizobia in Tryptone Yeast (TY) media at 28˚C (Beringer 1974). Where appropriate, media was supplemented with antibiotics at the following concentrations (μg ml^−1^): Spectinomycin (200), streptomycin (200), chloramphenicol (20), tetracycline (20) and nalidixic acid (75).

**Table 1:** Bacterial strains, plasmids and primers used in this study

Strain, plasmid or primer	Genotype^a^ and/description or sequence	N_2_ fixation effectiveness^b^	Reference
Strains			
CIAT899	*Rhizobium tropici* commercial inoculant for *P. vulgaris*; Nx^R^, Cm^R^, Sp^S^, Sm^R^	Effective	Martínez-Romero et al. (1991)
8002	*R. leguminosarum* isolated in Norfolk, UK	Effective	Johnston et al. (1982)
MUE254	*Escherichia coli* S17.1 pCAM131(mTn*5*SS*gusA*31) λ*pir*; Sm^R^_,_ Sp^R^_,_ Ap^R^	NA	Wilson et al. (1995)
ST18	*E. coli* S17.1 λ*pir* ∆*hemA*	NA	Thoma and Schobert (2009)
*P. vulgaris* rhizobia isolated from Kenya			
NAK103	*R. phaseoli* WSM4993	Effective	Mwenda et al. (2018)
NAK104	*R. paranaense* WSM4994	Effective	
NAK214	*Rhizobium* sp. WSM4997	Effective	
NAK220	*R. phaseoli* WSM4998	Effective	
NAK227	*Rhizobium* sp. WSM4999	Effective	
NAK239	*R. phaseoli* WSM5001	Effective	
NAK254	*Rhizobium* sp. WSM5003	Effective	
NAK284	*Rhizobium* sp. WSM5007	Effective	
NAK287	*R. phaseoli* WSM5008	Effective	
NAK288	*Rhizobium* sp. WSM5009	Effective	
NAK295	*Rhizobium* sp. WSM5010	Effective	
NAK299	*R. phaseoli* WSM5011	Effective	
NAK327	*Rhizobium* sp. WSM5014	Effective	
NAK378	*R. sophoriradicis* WSM5019	Effective	
NAK407	*R. phaseoli* WSM5021	Effective	
NAK458	*R. phaseoli* WSM5022	Effective	
NAK242	*R. phaseoli* WSM5002	Partially effective	
NAK266	*Rhizobium* sp. WSM5004	Partially effective	
NAK312	*Rhizobium* sp. WSM5012	Partially effective	
NAK315	*Rhizobium* sp. WSM5013	Partially effective	
NAK334	*Rhizobium* sp. WSM5016	Partially effective	
NAK358	*Rhizobium* sp. WSM5017	Partially effective	
NAK387	*R. sophoriradicis* WSM5020	Partially effective	
NAK120	*Rhizobium* sp. WSM4995	Poorly effective	
NAK231	*R. phaseoli* WSM5000	Poorly effective	
NAK332	*Rhizobium* sp. WSM5015	Poorly effective	
NAK368	*R. sophoriradicis* WSM5018	Poorly effective	
NAK210	*Rhizobium* sp. WSM4996	Ineffective	
**Plasmids**			
pJP2	Broad-host range plasmid carrying g*usA*; Tc^R^_,_ Ap^R^		Prell et al. (2002)
pMK-RQ-celBmNG	*P*_*tac-*_*celB-mNeonGreen* cloned in pMK; Km^R^		This work
pGM01	*P*_*tac-*_*celB-mNeonGreen* cloned at *Pst*I-*Xba*I site of pJP2, removing the *Pst*I-*Xba*I fragment carrying *gusA;* Tc^R^, Ap^R^		This work
**Primers**^**c**^			
CEK4	GGCCACGCGTCGACTAGTAC		Chun et al. (1997)
CEKGRNB1	GGCCACGCGTCGACTAGTACNNNNNNNNNNGCGCGC		This work
CEKGRNB2	GGCCACGCGTCGACTAGTACNNNNNNNNNNGCCGCC		This work
CEKGRNB3	GGCCACGCGTCGACTAGTACNNNNNNNNNYICCGCC		This work
CEKGRNB4	GGCCACGCGTCGACTAGTACNNNNNNBBBBNCCGCC		This work
GUS134	CTTGTAACGCGCTTTCCCAC		This work
WIL3	GAATGCCCACAGGCCGTCGAG		Wilson et al. (1995)
IE-F	CGATTGCCTTGAACTCACGG		This work
IE-R	CGAAGTAATCGCAACATCCGC		This work
OE-F	CTTGTAACGCGCTTTCCCAC		This work
OE-R	GAACGGCTCCAAGGAAGTGG		This work
PGM949F	GGAGAAGTACCGCAAGCTGT		This work
PGM1537R	CCGTTCTCTGGTAGATCGCC		This work

^a^ Ap, ampicillin; Cm, chloramphenicol; Km, kanamycin; Nx, nalidixic acid, Nm, neomycin; Sp, spectinomycin; Sm, streptomycin; Tc, tetracycline

^b^N_2_ fixation effectiveness as reported by Mwenda et al. (2018) and categorized as effective (mean shoot dry weight production > 75% that of CIAT899 inoculated plants), partially effective (between 75%-50%), poorly effective (between 50–25%) and ineffective (< 25%)

^c^ACGT-Standard nucleotides; N-any; Y-C or T; B-C or G or T; I-Inosine

### Marking of CIAT899 with *gusA* reporter gene

*R. tropici* CIAT899 was marked with mTn*5*SS*gusA*31, a mini-transposon carrying *gusA* expressed by the symbiotically active *nifH* promoter (Wilson et al. 1995). The mini-transposon was transferred from *E. coli* S17.1 strain (MUE254) by bi-parental mating into CIAT899 (Reeve et al. 2016) and transconjugants selected on TY media supplemented with spectinomycin, streptomycin, chloramphenicol and nalidixic acid. Selected transconjugants were assessed for growth rate, N_2_ fixation efficiency and competitiveness for nodulation on *P. vulgaris*.

The integration site of mTn*5*SS*gusA*31 in CIAT899 transconjugants was identified by a three-step nested PCR and subsequent sequencing approach. In the first step, amplification of single stranded DNA from *gusA* was achieved with primer GUS134 (Table 1) and 5 ng μl^−1^ genomic DNA, using cycling conditions of 94 °C for 2 min, followed by 10 cycles of 94 °C for 30 s, 60 °C for 30 s and 70 °C for 90 s. The reaction products were diluted fivefold with water and a 1 μl aliquot used as template for the second PCR, primed with GUS134 paired separately with CEKGRNB1, CEKGRNB2, CEKGRNB3 or CEKGRNB4 (Table 1). The products of this second round of PCR were again diluted fivefold, and 1 μl aliquots used as template for the third PCR, primed with WIL3 and CEKG4. Cycling conditions for the second and third round of PCR were as per Chun et al. (1997). The products of the third round of PCR were separated in 1% (w/v) agarose, excised, purified and Sanger sequenced using WIL3 and CEKG4 primers. Homology searches and sequence comparisons were carried out using the NCBI BLASTN search algorithm and transposon sequences distinguished from chromosomal sequences. The identified insertion site was further confirmed by PCR and sequencing of the DNA at both ends of the inserted mini-transposon using primer pairs IE-F with IE-R and OE-F with OE-R. The N_2_ fixation ability of CIAT899*gusA* was compared to that of wild-type CIAT899 on *P. vulgaris* cv. Tamu 42 days post-inoculation, as previously described (Mwenda et al. 2018).

### Marking strains with the *celB* reporter gene

All 30 rhizobial strains used in this study (Table 1) were marked with a *celB-*expressing plasmid. The *celB*-expressing plasmid consisted of a *celB* and *mNeonGreen* cassette cloned into the stable broad-host range plasmid pJP2 (Prell et al. 2002). The insert was designed in Geneious (Biomatters Ltd, NZ), using *celB* (Sessitsch et al. 1996) and *mNeonGreen* (Shaner et al. 2013) sequences obtained from GenBank (GenBank Accession 11847805 and AGG56535.1). The sequences were fused, silent mutations introduced to remove XbaI and PstI restriction sites, and the constitutive *tac* promoter and terminal XbaI and PstI restriction sites added. After *in silico* assembly, the construct was chemically synthesised (GeneArt, Thermo Fisher Scientific) and cloned into the PstI-XbaI site of pJP2, after excision of *gusA*, resulting in the constitutive expression vector pGM01. This vector was introduced into *E. coli* ST18 (Thoma and Schobert 2009) by electroporation and into rhizobia by bi-parental conjugation (Reeve et al. 2016). Transconjugants were selected on TY media supplemented with tetracycline, verified first by PCR with primers PGM949F (binding to the *oriV* origin of replication in pGM01) and PGM1537R (binding to *celB*) and expression was subsequently confirmed by a β-galactosidase assay (Sessitsch et al. 1996), modified to include a heat-inactivation step (70 °C for 45 min) to inactivate endogenous β-galactosidase activity. The *celB*-marked strains were subsequently assessed for similarity to wildtype colony morphology and, for a subset, growth rate and competitiveness for nodulation of *P. vulgaris* was also assessed.

### Root staining and scoring of nodule occupants

GUS staining and clearing of roots was performed as described previously (Wilson et al. 1995) with the exception that roots were first vacuum-infiltrated for 30 min in the GUS staining buffer containing 200 µg mL^−1^ of 5-bromo-4-chloro-3-indolyl-β-D-glucuronic acid (X-Glc) before incubation on a shaker (200 rpm) at 37 °C for 24 h. The CelB staining process was the same as that for GUS, but with 200 µg mL^−1^ 5-Bromo-6-chloro-3-indolyl β-D-galactopyranoside (Magenta-Gal) substituted for X-Glc. To double stain, roots were first stained with X-Glc, incubated at 70 °C for two hours to destroy endogenous β-galactosidases, then stained with Magenta-Gal. Finally, roots were de-stained in 4% (w/v) sodium hypochlorite and rinsed thoroughly. Nodules formed by CIAT899*gusA* stained blue while those formed by *celB*-marked strains were magenta. Mixed nodules had various patterns of both colours.

### Competition assays

To measure the competitiveness of strains, *P. vulgaris* cv. Kenya Tamu was co-inoculated with suspensions of liquid cultures of *celB*-marked strains and the CIAT899*gusA* reference strain. First, cells in logarithmic phase were harvested by centrifugation and washed free of antibiotics with sterile deionised water and resuspended to 1 × 10^4^ cells ml^−1^. Each of the *celB*-marked strains was pre-mixed with CIAT899*gusA* (at 1:1 volume ratio) and one ml of this suspension inoculated onto surface-sterilised *P. vulgaris* seeds and sown into pots containing steam-sterilised sand. At the time of seed inoculation, viable cell counts and the proportion of each strain in the bacterial suspensions were confirmed by the Miles and Misra technique (O’Hara et al. 2016). Treatments had three pot replicates, each containing two seeds. Plants were maintained in a naturally-lit glasshouse as described previously (Mwenda et al. 2018), harvested after 21 days following inoculation and roots double-stained with X-Glc and Magenta-Gal. Competitiveness index (CI) was then calculated as follows: *CI of Y* = $(nodulesY/nodulesX)/(cfuY/cfuX)$, where *cfuY* and *cfuX* are the number of colony forming units of co-inoculated strains Y and X per ml of inoculant suspension applied to plants, and nodules*Y* and nodules*X* denotes the number of nodules occupied by the respective strains.

### Rhizosphere colonization assays

Four *celB*-marked strains (NAK120*celB*, NAK210*celB*, NAK287*celB* and CIAT899*celB*) plus CIAT899*gusA* were cultured in TY broth until mid-logarithimic phase (OD_600_ = 0.5) and a 1 ml aliquot of each was centrifuged (10,000 g, 1 min), washed and resuspended in sterile water. Each suspension was serially diluted to give a final viable cell count of 2 × 10^3^ cells ml^−1^, and then each *celB*-marked strain was individually mixed in a 1:1 ratio with the CIAT899*gusA* suspension. For each combination, a 1 ml aliquot was removed for viable cell counts on TY agar with tetracycline (for enumeration of *celB* strains) and TY agar with spectinomycin (for enumeration of CIAT899*gusA*) and a further 1 ml aliquot was inoculated onto surface-sterilised pre-germinated seeds of *P. vulgaris* as described above. Treatments were replicated in eight pots, with two plants per pot which were thinned to one plant per pot at emergence. Seven days after inoculation, four plants from each treatment were carefully removed from their pots and roots gently shaken to remove excess soil. The roots, together with lightly adhering soil, were transferred to 50 ml tubes and resuspended in sterile water before shaking for 30 min at medium speed on an Analite wrist shaker (Ananlite Pty, Australia), after which viable cell counts were determined (O’Hara et al. 2016). Plants in the remaining pots were harvested 17 days after inoculation and roots double stained with X-Glc and Magenta-Gal and counted as described above.

### Competition for nodulation of *P. vulgaris* between seed- and soil-borne resident strains

Sterile soil was separately inoculated with NAK120*celB*, NAK210*celB*, NAK287*celB* and CIAT899*celB* at 10^2^ and 10^5^ cells.g soil^−1^ to establish these rhizobia as soil-borne resident strains. To achieve this, bacteria were cultured to mid-log phase in TY broth and aliquots containing 2.72 × 10^5^ or 2.72 × 10^8^ cells re-suspended in 80 ml of sterile water. The suspensions were individually mixed into 3.2 kg steam-sterilised soil resulting in 10^2^ and 10^5^ rhizobial cells.g soil^−1^. Pots were covered with plastic wrap and kept in a shaded glasshouse maintained at 22 °C for four days before sowing. Viable cell counts of rhizobia were performed at sowing, 10 days and 30 days post sowing, from a depth of 5–10 cm by the Miles and Misra technique on TY medium supplemented with tetracycline (20 µg mL^−1^).

*P. vulgaris* cv. Kenya Tamu seed was inoculated at two rates with CIAT899*gusA* and sown into soils containing the pre-established soil-borne resident strains. Firstly, an inoculant carrying 10^9^ cells.g peat^−1^ of CIAT899*gusA* was prepared using standard techniques (Yates et al. 2016) and cured for 10 days at 28 °C. This inoculant was diluted 100-fold by mixing with sterile peat and adjusted to 25% moisture with deionised water, resulting in a second inoculant carrying 10^7^ cells.g peat^−1^. Surface-sterilised seed (Mwenda et al. 2018) was then inoculated with each peat preparation, using established techniques (Yates et al. 2016), with 40% (w/v) gum arabic as the sticker, to obtain a low and high inoculation rate. Rhizobia on seeds were counted by the Miles and Misra method. Two seeds inoculated with CIAT899*gusA*-containing peat, were sown into each pot containing the pre-established soil-borne resident strains. For the uninoculated treatment, uninoculated surface-sterilised seed was sown into pots with steam sterilised soil. Plants were maintained with N-free nutrient solutions and sterile deionised water as described previously (Mwenda et al. 2018) and harvested after 30 days, roots double-stained with X-Glc and Magenta-Gal, and nodules counted.

### Data analysis

Data were analyzed by means with standard errors, Pearson’s correlation and where applicable, an analysis of variance (ANOVA) using SPSS version 22 (IBM Corp, released 2013). ANOVA was preceded by a test for normality and equal variances (Levene’s test). Tukey’s HSD was then used when ANOVA was found to be significant (P ≤ 0.05).

## Results

### Construction and validation of marked strains

To determine the competitiveness of *R. tropici* CIAT899, a highly effective N_2_-fixing symbiont of *P. vulgaris* and a common inoculant for this legume worldwide (Bala et al. 2011; Hungria et al. 2003), to *P. vulgaris*-nodulating rhizobia from Kenya, a differential staining approach was used to identify nodule occupants. The reference strain CIAT899 was marked with *gusA* (β-glucuronidase), while the strains from Kenya were marked with a plasmid-borne *celB*, encoding a thermostable β-glucosidase. The *gusA* gene under the control of the *nifH* promoter, which drives the expression of the genes encoding the nitrogenase complex, was transferred into CIAT899 using a mini-transposon (mTn*5*SS*gusA*31), which inserted 6-bp from the 3' end of universal stress protein A gene (*uspA*, locus tag RTCIAT899_CH07670 in the CIAT899 chromosome). In TY liquid culture, the mean generation time of *gusA*-marked CIAT899 (CIAT899*gusA*) did not differ significantly (P > 0.01) to that of wild-type CIAT899 [112 ± 5 min (standard error of the mean) and 111 ± 7 min, respectively]. Similarly, *P. vulgaris* plants inoculated with CIAT899*gusA* and cultivated in N-free conditions, yielded mean shoot dry weights that were indistinguishable from wild-type CIAT899 inoculated plants (3.54 ± 0.39 g vs 3.28 ± 0.23 g; P > 0.01). When co-inoculated onto plants in a 1:1 ratio of CIAT899*gusA* and wild-type CIAT899, 50.3% of the nodules were occupied by CIAT899*gusA* (stained blue with X-Glc) and 49.7% by the wild-type CIAT899. This indicates that *gusA*-marked CIAT899 is as competitive at nodulating *P. vulgaris* as CIAT899, so insertion of *gusA* into *uspA* in CIAT899*gusA* did not compromise the free-living growth, N_2_ fixation or competitiveness of this strain.

**Fig. 1:**
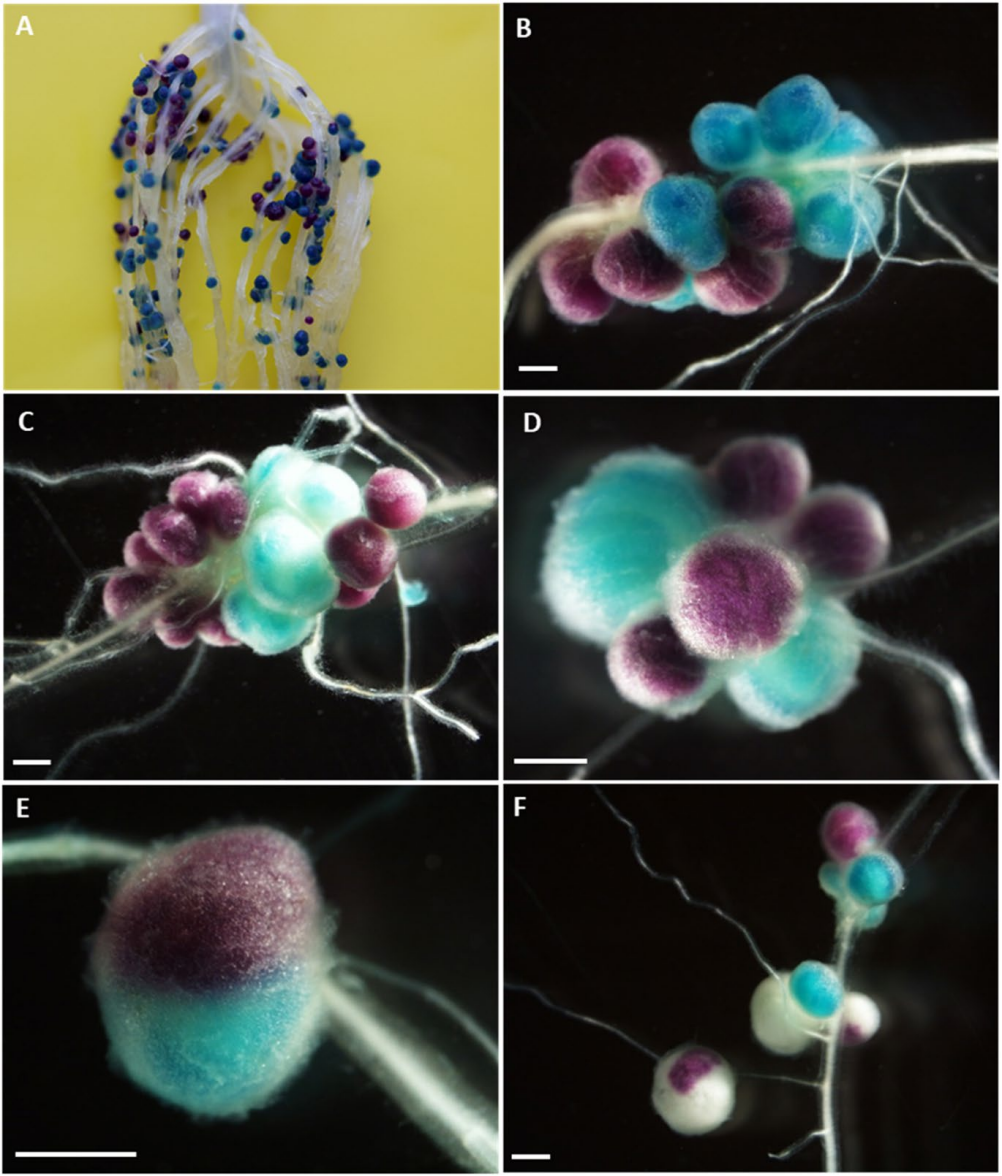
*P. vulgaris* root nodules stained with X-Glc (blue) and Magenta-Gal (magenta) co-inoculated with (**A** and **B**) CIAT899*gusA* (blue nodules) or CIAT899*celB* (magenta nodules). (**C**) NAK104 and CIAT899*gusA* (**D**) NAK387 and CIAT899*gusA*. (**E**) Mixed nodule containing both CIAT899*gusA* and CIAT899*celB.* (**F**) *P. vulgaris* root nodules formed following inoculation with CIAT899*gusA* and NAK407*celB*, showing white nodules formed by the loss of the *celB*-harboring plasmid pGM01 for NAK407. Scale bar is 1 cm

Plasmid pGM01, carrying *celB* under the control of the constitutive *tac* promoter, was conjugated into the 28 *P. vulgaris*-nodulating Kenyan *Rhizobium* strains, plus the commercial inoculant CIAT899 and *R. leguminosarum* sv. phaseoli 8002, a well-studied and effective *P. vulgaris* nodulating strain from the UK (Mwenda et al. 2018; Johnston et al. 1982). The growth rate of five selected strains carrying pGM01 (CIAT899, NAK120, NAK210, NAK239, NAK103) in TY broth (without antibiotic selection) did not differ to the growth rate of the respective unmarked wild-type strains, indicating the presence of the plasmid did not affect strain growth. Furthermore, when a 1:1 ratio of CIAT899*gusA* and CIAT899*celB* were co-inoculated onto *P. vulgaris* and stained with X-Glc and Magenta-Gal, both blue and magenta nodules were observed (Fig. 1A and B) in equal numbers (48 ± 5.6 mean number blue nodules.plant^−1^ vs 46 ± 6.5 mean number magenta nodules.plant^−1^), indicating that both marked strains were equally competitive and there was no impact on competition from harboring a chromosomal or plasmid-borne chromogenic marker gene.

### Nodulation competitiveness

Next, we determined how capable each of the 30 *celB*-marked strains were at nodulating *P. vulgaris* when approximately 10^4^ cells of each strain were individually co-inoculated at a 1:1 ratio on plants with CIAT899*gusA*. On post-harvest staining, 23 strains yielded nodules that were either blue, indicating the presence of CIAT899*gusA* in the nodule, or magenta, indicating the presence of the *celB*-marked strain (Fig. 1C and D). In 9% of nodules across treatments, both blue and magenta staining of a single nodule was observed, suggesting mixed infection of the nodule by both *gusA* and *celB* marked strains (Fig. 1E). For seven strains (NAK315, NAK334, NAK368, NAK407, NAK287, NAK103 and NAK299) carrying the *celB* marker, partially unstained or completely unstained nodules were observed following treatment with Magenta-Gal (Fig. 1F). These unstained nodules were observed alongside blue stained nodules, indicative of the presence of CIAT899*gusA*. The lack of staining, or partial staining, suggested instability of the *celB*-harboring plasmid (pGM01) in these strains. Re-isolation from stained nodules to confirm loss of plasmid from these strains *in planta* was not possible, as the CelB staining procedure requires heat-treatment to inactivate background β-galactosidase activity. Therefore, three of the seven strains (NAK287*celB*, NAK334*celB* and NAK407*celB*) and three strains that did not show unstained or partially stained nodules (CIAT899*celB*, NAK120*celB* and NAK210*celB*) were selected and the stability of pGM01 in these strains was assessed in free-living culture in the absence of antibiotic selection. After 20 generations, 100% of the CIAT899, NAK120 and NAK210 cells tested retained pGM01. In contrast, for the three strains that showed evidence of unstained nodules, all showed loss of the plasmid, with only 50% of NAK334, 78% of NAK287 and 72% NAK407 cells retaining the plasmid after 20 generations. This therefore indicates that loss of the plasmid *in planta* was the likely cause of partially or completely unstained nodules from these seven strains. Consequently, unstained nodules from plants co-inoculated with these seven strains were scored as magenta.

**Fig. 2:**
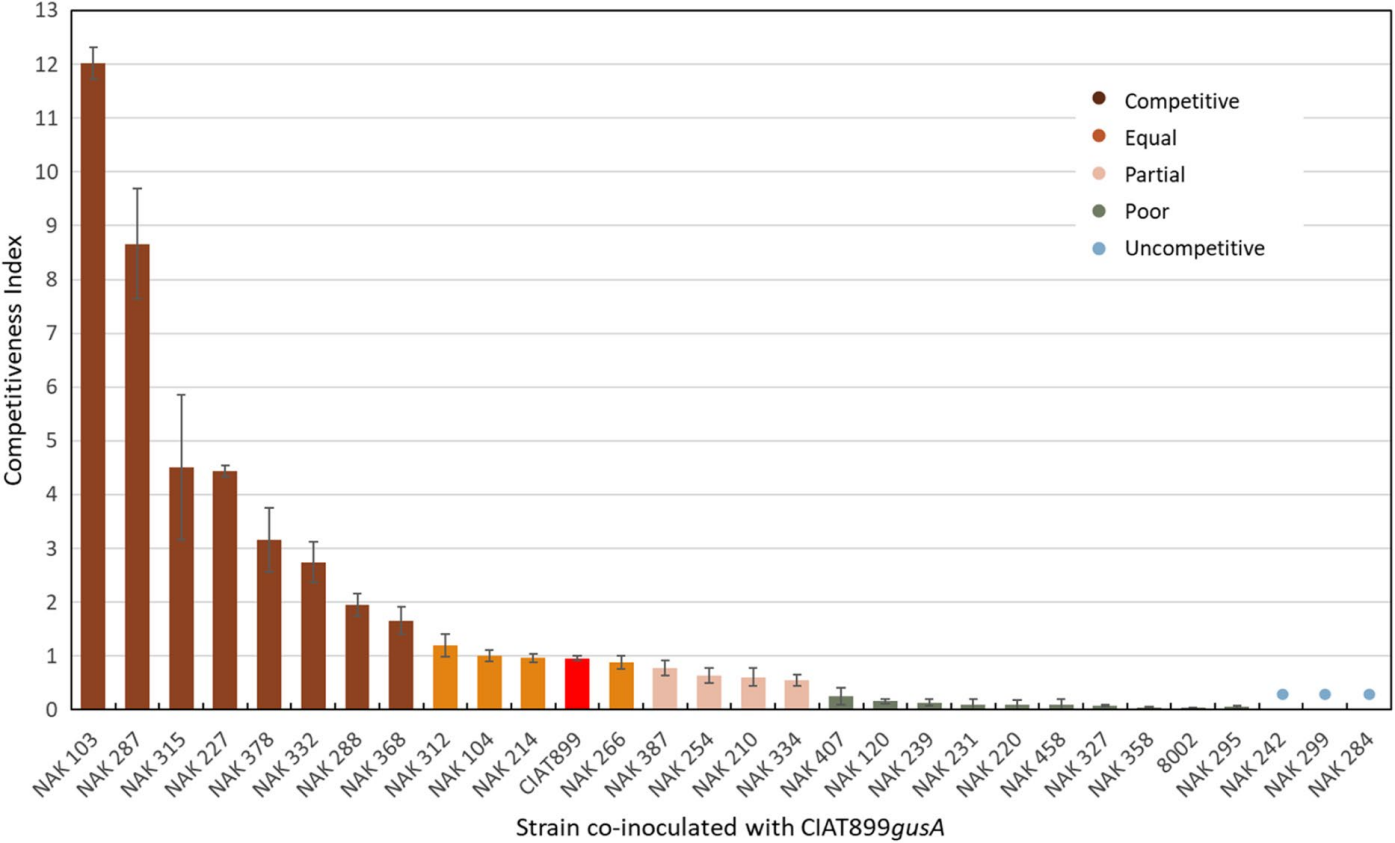
Competitiveness of 30 *P. vulgaris*-nodulating strains carrying *celB* when co-inoculated with CIAT899*gusA*. Strain competitiveness index was calculated from the ratio of nodules occupied by the test (i.e. *celB*-marked strain) and reference (CIAT899*gusA*) strains, normalized for the proportion of viable cells of each strain inoculated onto plants. Strains were classed as more competitive (ratio > 1.2), equally competitive (1.2 to 0.8), partially competitive (0.8 to 0.5) and poorly competitive (< 0.5). NAK242, NAK299 and NAK284 did not nodulate *P. vulgaris* when co-inoculated with CIAT899 and were therefore classed as uncompetitive. CIAT899*celB*, which was equally competitive with CIAT899*gusA*, is denoted by a red column

Strain competitiveness was calculated from the ratio of nodules occupied by the test (i.e. *celB*-marked strain) and reference (CIAT899*gusA*) strains, normalized for the proportion of viable cells of each strain inoculated onto plants. Mixed nodules were excluded from this calculation. Analysis of competitiveness for nodulation among the strains showed a wide range of phenotypes compared to CIAT899*gusA* (Fig. 2). Eight of the strains were more competitive (competitiveness > 1.2) at nodulating *P. vulgaris* than CIAT899, with the most competitive strain being NAK103 yielding 12-fold more nodules on this host. Five strains, including CIAT899 harboring the *celB* plasmid, were equally competitive (competitiveness between 0.8 and 1.2) at nodulating *P. vulgaris*. Four strains (NAK387, NAK254, NAK210, NAK334) were partially competitive compared to CIAT899*gusA*, yielding ratios of between 0.8 and 0.5. A further ten strains were poorly competitive, yielding fewer than half the number of nodules on *P. vulgaris* compared to the reference strain (competitiveness < 0.5). Three strains (NAK242, NAK299 and NAK284) did not nodulate *P. vulgaris* when co-inoculated with CIAT899 and were therefore categorized as uncompetitive.

### No correlation between competitiveness and symbiotic effectiveness

**Fig. 3:**
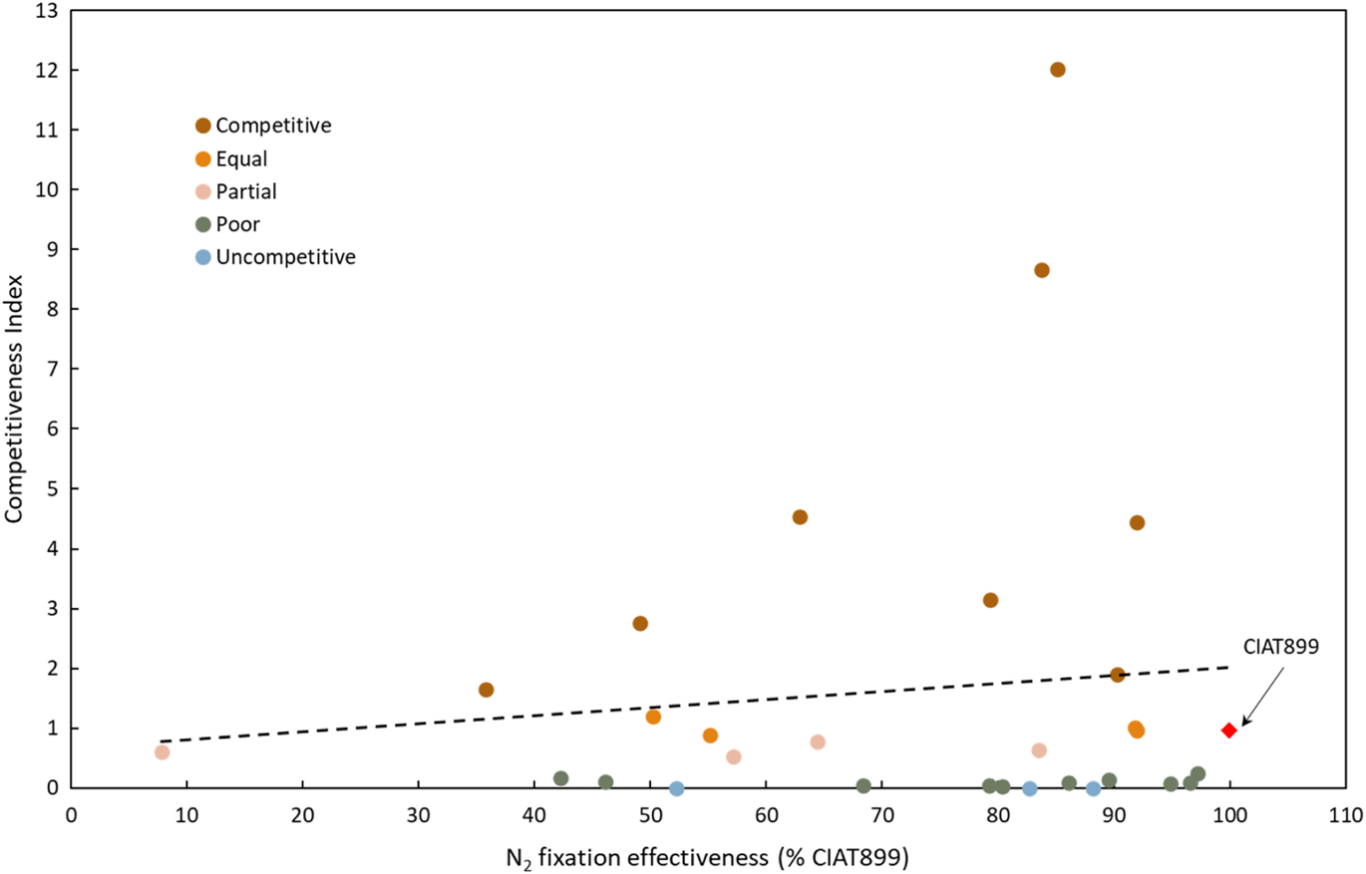
Correlation of the competitiveness of 30 *P. vulgaris* nodulating strains with their symbiotic effectiveness. Data for N_2_ fixation effectiveness from Mwenda et al. (2018) expressed as a percentage of biomass achieved by plants inoculated with the test strain compared to CIAT899. Strain competitiveness index was calculated as indicated earlier. CIAT899 (defined as 100% effective and equally competitive) denoted by a red diamond. Dotted line denotes the linear correlation between the two traits, with r^2^ = 0.0114

In a previous study, the capacity of 28 Kenyan strains plus 8002 to fix N_2_ with *P. vulgaris* was compared to CIAT899 (Mwenda et al. 2018). To investigate whether a link between competitiveness and effectiveness of these strains existed, we analyzed the correlation between their observed competitiveness and N_2_ fixation capacity, expressed as a percentage of biomass achieved by plants inoculated with the test strain compared to CIAT899. We found that competitiveness did not correlate to effectiveness for N_2_ fixation (r^2^ = 0.0114) (Fig. 3). For example, of the 17 strains that were effective N_2_ fixers (> 75% of N_2_ fixed by CIAT899), only seven were either more competitive or equally competitive with CIAT899, while a further seven strains were poorly competitive, which included the least competitive strains NAK295 and 8002. The two remaining effective strains (NAK299 and NAK284) were uncompetitive (i.e. did not nodulate *P. vulgaris*). These data indicate that the traits of competitiveness and symbiotic N_2_ fixing effectiveness are not correlated in the strains analyzed.

### Rhizosphere competence correlates with nodule occupancy

We next investigated the role of rhizosphere competence in nodulation outcomes when strains were co-inoculated with CIAT899*gusA* onto *P. vulgaris*. Four *celB*-harboring strains were chosen, representing strains that were more competitive (NAK287), equally competitive (CIAT899), partially competitive (NAK210) and poorly competitive (NAK120). Strains were co-inoculated with CIAT899*gusA* at ~ 10^3^ cells seed^−1^, yielding approximately equal proportions of each strain at time of inoculation (Table 2). For the CIAT899*celB* and CIAT899*gusA* treatment, this equal proportion of strains was maintained in host rhizospheres sampled seven days after inoculation and in nodules on *P. vulgaris* plants harvested at 17 days post inoculation (Table 2), which supports there being no differences in competitiveness in these two strains, as was previously observed (Fig. 2). For NAK210, shown to be partially competitive from the earlier co-inoculation experiment with mature nodules at 21 days post inoculation, the proportion of cells declined from 43% at inoculation to 2% in the rhizosphere, and this low proportion was maintained through to nodulation, with only 5% of nodules occupied by this strain. While a similar, but less severe, decline in rhizosphere cell numbers was observed for NAK120 (41% to 14%), this strain did not occupy any *P. vulgaris* nodules. Therefore, both partially (NAK210) and poorly (NAK120) competitive strains were outcompeted by CIAT899 in the rhizosphere, and this was mirrored in later nodulation outcomes.

**Table 2:** Inoculation rates, rhizosphere populations at day 7 and nodule occupancy at day 17 following co-inoculation of CIAT899*gusA* separately with CIAT899*celB*, NAK287*celB*, NAK210*celB* or NAK120*celB* onto *P. vulgaris* seeds^a^

	Inoculation (cells.seed^−1^)			Rhizosphere^b^ (× 10^5^ cells.g rhizosphere matter^−1^)			Nodulation (Nodule number.plant^−1^)			
Test strain	Test strain	CIAT899 reference	Proportion^c^ (%)	Test strain	CIAT899 reference	Proportion (%)	Test strain	CIAT899 Reference	Proportion (%)	Mean nodule number
CIAT899	824 ± 11	1049 ± 17	44|56	60 ± 4	75 ± 10	45|55	66 ± 9	84 ± 16	44|56	150
NAK287	837 ± 9	1063 ± 6	44|56	18 ± 6	117 ± 20	14|86	66 ± 10^c^	66 ± 11	50|50	132
NAK210	857 ± 55	1137 ± 61	43|57	1.8 ± 0.5	120 ± 20	2|98	4 ± 2	83 ± 15	5|95	87
NAK120	760 ± 21	1073 ± 60	41|59	20 ± 6	128 ± 30	14|86	0	88 ± 29	0|100	88

^**a**^ Each test strain (harboring *celB*) was co-inoculated with reference strain CIAT899 (harboring *gusA*) with *P. vulgaris* seed in eight pots. The mean number of viable cells in test and reference strains at inoculation (n = 8), within the rhizosphere (n = 4) and the number of test (i.e. magenta) and reference (blue) nodules at 17 days post-inoculation (n = 4) was determined. The proportion of test vs reference cells (at inoculation and in the rhizosphere) and nodules was calculated as a percentage

^**b**^Rhizosphere numbers are given per g of rhizosphere matter (roots and adhering soil)

^c^Unstained nodules were counted as occupied by NAK287*celB* as they were inferred to have arisen from infection by NAK287 cells that had lost pGM01

Cell numbers per seed, mean viable cell numbers and mean nodule numbers are shown ± standard errors of the means

A decline in the proportion of NAK287 from 44% at inoculation to 14% in the rhizosphere was also observed, with this proportion then increasing to 50% occupancy in *P. vulgaris* root nodules (Table 2). As with the previous co-inoculation experiment, unstained nodules were also observed on plants of this treatment (caused by the instability of pGM01 plasmid in NAK287), so all unstained nodules from this treatment were scored as harboring NAK287. However, strain numbers in the rhizosphere were determined by addition of antibiotics to media to distinguish between CIAT899*gusA* (streptomycin resistant) and *celB*-harboring strains (tetracycline resistant) strains. The loss of plasmid pGM01 from some of the NAK287 cells would therefore mean the true density of NAK287 in the rhizosphere would be higher than recorded, as NAK287 cells that had lost pGM01 could not be enumerated on tetracycline plates. These data therefore suggest that NAK287 was likely able to maintain relatively equal numbers in the rhizosphere, leading to competitive nodulation of *P. vulgaris* compared to CIAT899.

### Inoculation success in soils with established resident rhizobia

Having determined the competitiveness phenotypes of *P. vulgaris* nodulating rhizobia, we next investigated the outcome of strain competition in a system designed to mirror inoculation of *P. vulgaris* in the field, where a seed-borne inoculant is introduced at the time of sowing, into a soil with a pre-existing rhizobial population. To create the pre-existing populations, we chose the same test strains as the rhizosphere experiment (i.e. NAK287, CIAT899, NAK210 and NAK120) and inoculated them separately into sterile soil at 10^2^ and 10^5^ rhizobia.g soil^−1^. After a week in soil devoid of organic matter, added nutrients and host plants, these densities increased to (6.2 ± 0.7) × 10^4^ and (1.8 ± 0.4) × 10^6^ cells.g soil^−1^, respectively, creating two rhizobial densities similar to those often reported in soils where *P. vulgaris* is cultivated. *P. vulgaris* seeds inoculated with CIAT899*gusA* at low [(7.4 ± 0.4) × 10^4^ cells.seed^−1^] and high [(6.6 ± 0.4) × 10^6^ cells.seed^−1^] rates, representing rates below and above the recommended 10^5^ cells seed^−1^ (Bullard et al. 2005; Lupwayi et al. 2000), were sown into pots containing these pre-existing rhizobial populations. The resultant plants were harvested 30 days after sowing and nodule occupants assessed using the dual marker system.

**Fig. 4:**
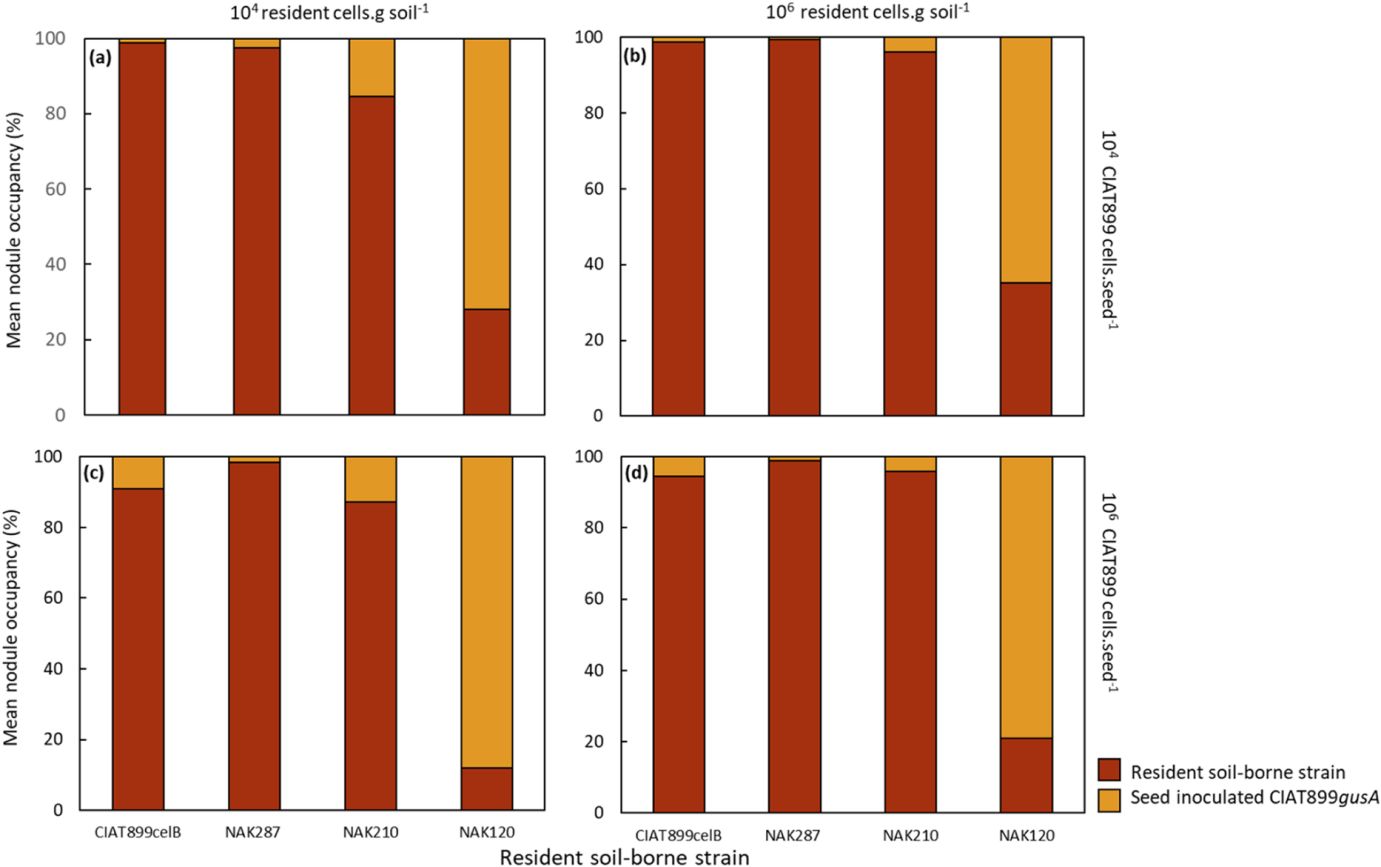
Percentage of *P. vulgaris* nodules occupied by seed-borne CIAT899*gusA* versus percentage occupied by resident soil-borne CIAT899 (equally competitive), NAK 287 (more competitive), NAK210 (partially competitive) or NAK120 (poorly competitive), each individually harboring *celB* on pGM01. The seed-borne strain was applied at 7.4 ± 0.4 × 10^4^ cells seed^−1^ and 6.6 ± 0.4 × 10^6^ cells seed^−1^, while the soil-borne strain densities were 6.2 ± 0.7 × 10^4^ and 1.8 ± 0.4 × 10^6^ cells g^−1^ of soil at planting. Data are means of eight plant replicates per treatment

At both low and high seed and soil inoculation rates, seed-borne CIAT899*gusA* was outcompeted in soils with resident populations of *celB*-marked CIAT899 (equally competitive to CIAT899*gusA*), with 91% or more of nodules occupied by the strain resident in the soil (Fig. 4). A similar outcome was observed when NAK287 (more competitive) and NAK210 (partially competitive) were inoculated into soil, with 98% or more (for NAK287) and 85% or more (for NAK210) of nodules occupied by these resident strains. In contrast, when the poorly competitive strain NAK120 was resident in the soil at high densities (i.e. 10^6^ cells.g soil), only 35% and 21% of nodules on *P. vulgaris* were formed by this strain at both low and high CIAT899*gusA* seed inoculation rates (Fig. 4b and d). This proportion of nodules occupied by NAK120 dropped to 28% and 12% when the same rates of seed-borne CIAT899*gusA* were inoculated at low NAK120 soil densities (i.e. 10^4^ cells.g soil^−1^). Therefore, these results indicate that in soils with rhizobial densities of 10^4^–10^6^ cells.g soil^−1^, superior inoculation rates are inadequate to overcome nodulation of the legume by strains resident in the soil, unless the resident strains compete poorly with the inoculant strain.

## Discussion

A wide range of competitiveness phenotypes were observed in the 28 *P. vulgaris*-nodulating rhizobia isolated from Kenya, with only 27% of strains more competitive at nodulating *P. vulgaris* than CIAT899. This result is consistent with the long-standing use of CIAT899 in Africa and South America as a competitive and highly effective inoculant strain for *P. vulgaris*, with the strain also tolerant to high temperature and soil acidity (Bala et al. 2011; Hungria et al. 2000, 2003). Crucially, the competitiveness of the strains examined in this work did not correlate with strain effectiveness, with many instances of highly effective strains being poorly competitive, including the well-studied and highly effective *P. vulgaris* reference strain *R. leguminosarum* sv. phaseoli 8002, while some strains were even unable to nodulate *P*. vulgaris when co-inoculated with CIAT899. Only five strains (NAK103, NAK287, NAK227, NAK288 and NAK378) were more competitive than CIAT899 and capable of fixing N_2_ effectively with *P. vulgaris*. This emphasizes that competitiveness and effectiveness are separate genetic traits (Mendoza-Suarez et al. 2020) and reinforces the importance of measuring both characteristics when evaluating strains for suitability as inoculants.

Plasmid pGM01 proved an effective vector to express the *celB* reporter gene, and the plasmid was easily conjugated into all target strains. (Sanchez-Canizares and Palacios 2013; Sessitsch et al. 1996). While the plasmid was stable in free-living conditions under tetracycline selection, it was unstable in a small number of strains *in planta* (7 strains out of 30) and in a subset of these strains tested in the absence of antibiotic selection. pGM01 is derived from the expression vector pJP2, which itself was constructed from the stable broad-host range parent plasmid RK2 (Prell et al. 2002). Plasmids pJP2 and pGM01 harbor the *parCBA* plasmid partitioning system and the *parDE* toxin-antitoxin plasmid stability system, and pJP2 has been widely used as a stable expression system across a range of *Alphaproteobacteria* (Haskett et al. 2018; Mendoza-Suarez et al. 2020; Mulley et al. 2011; Prell et al. 2009; Tett et al. 2012). Why pGM01 was unstable in some strains assessed in this study is unclear, although it has previously been noted that the level of stability provided by *parCBA* can be strain dependent (Easter et al. 1998). Given that the 28 Kenyan strains investigated here represent at least six species of *Rhizobium* (Mwenda et al. 2018), it is possible that different expression levels of plasmid stability determinants in some of these diverse backgrounds could have resulted in plasmid loss.

Previous studies using *gusA* and *celB* dual markers have typically delivered these reporter genes via mini-transposons (Sessitsch et al. 1996) or via insertion into the bacterial genome by homologous recombination (Sanchez-Canizares and Palacios 2013). While these approaches generate stable marked strains, they can also generate variants with altered symbiotic phenotypes or require detailed genome sequence analysis to perform, which tends to limit their applicability to smaller number of strains. In contrast, although pGM01 appears unstable in some strain backgrounds, a majority of test strains in this study were able to stably maintain pGM01, making the plasmid a useful tool for dual strain competition experiments where screening of a relatively large numbers of strains is required.

Strains CIAT899, NAK287, NAK210 and NAK120 differed in their ability to colonize the rhizosphere of *P. vulgaris* when co-inoculated with CIAT899, with the proportion of nodules occupied by the strains strongly mirrored by the proportion of cells in the rhizosphere. Only NAK287 did not show this trend, however it is highly likely this was due to the instability of pGM01 in this strain, rather than an actual decrease in numbers of NAK287 in the soil. The ability of strains to colonize and thrive in the rhizosphere is related to a range of factors, including the survival of inoculant strains on seed (Deaker et al. 2004), survival and growth of strains under prevalent environmental conditions such as pH (Anyango et al. 1998), strain growth rates (Li and Alexander 1986), tolerance to microbial antagonism (Mrabet et al. 2006), chemotaxis and motility (Cooper 2007), root attachment (Janczarek et al. 2015), and ability to metabolize plant root exudates (Streit et al. 1992). In fact, a recent INSeq analysis of *Rhizobium leguminosarum* bv. viceae 3841 in the *Pisum sativum* rhizosphere showed 170 genes were required for rhizosphere growth (Wheatley et al. 2020). It is therefore likely that one or more genetic differences in rhizosphere competence between strains tested in this study may have caused a relatively enhanced ability of one strain to nodulate *P. vulgaris* ahead of another. Rhizosphere competence appears to be a significant factor in nodulation outcomes and therefore critical when considering new inoculant strains.

When seed inoculated CIAT899 was sown into soils with pre-existing rhizobial populations of 10^4^ or 10^6^ cells.g soil^−1^, nodulation of *P. vulgaris* was dominated in most cases by rhizobia resident in the soil at both low and high inoculation densities. Only when the resident strain was poorly competitive (NAK120), was seed-borne CIAT899 able to occupy a majority of root nodules. Most strikingly, when CIAT899 was both the seed- and soil-borne resident strain, more than 95% of nodules were formed by CIAT899 pre-existing in the soil. This indicates that the resident strain has a strong advantage in nodulating the target legume and only when the soil harbors a very poorly competitive strain, can the seed-borne inoculant dominate in the nodule. A likely key difference between seed and resident soil rhizobia was their physiological state, in that the inoculant strain was shifted from a relatively nutrient-rich and high moisture environment of the peat to the nutrient-poor and drier environment of the soil. In contrast, the resident strain had increased in number over a seven-day period in the soil prior to sowing with the inoculant-carrying seed and was presumably habituated to this environment. This physiological difference between inoculant and resident strains would likely lead to large differences in the ratio of the two strains during seedling germination. In fact, in a system which compared nodulation of *Glycine max* by two isogenic and equally competitive antibiotic resistant derivatives of *Bradyrhizobium japonicum* E11, the resident strain outnumbered the seed inoculated strain in the root infection zone four- to seven-fold (López-García et al. 2002). This type of position-dependent numerical advantage, which sees the resident strain dominate the infection zone of the host legume, would represent a significant hurdle for an inoculant strain to overcome, unless resident rhizobia are poorly competitive, such as was shown with NAK120 in this work.

*P. vulgaris* is often cultivated in soils with between 10^4^ to 10^6^ rhizobia.g soil^−1^ (Alberton et al. 2006; Anyango et al. 1998; Hungria et al. 2000; Thies et al. 1991b), and the numbers of cells in the soil environment tested in this study reflect these population sizes. Numerous studies investigating the effect of soil rhizobia population size on inoculation success in the field have shown that nodulation can be increased following inoculation in soils with 10^3^ rhizobia.g soil^−1^ (Bergersen 1970; Hungria et al. 2000; Kyei-Boahen et al. 2017), 10^4^ and 10^5^ rhizobia.g soil^−1^ (Hungria et al. 2003; Vlassak et al. 1996) but also that nodulation by the inoculant can be inhibited in soils with as few as 10^2^ rhizobia.g soil^−1^ (Singleton and Tavares 1986) or even less (Thies et al. 1991a). Genotypic variation of rhizobia in these different soil environments is the likely cause of these different responses to inoculation. Thus, if soils are composed mainly of strains that are highly competitive for nodulation of the target legume, it can be expected that even at relatively low numbers, they would dominate the inoculant strain. Conversely, soils composed predominantly by poorly competitive strains would present little barrier to inoculation response.

Five of the 28 Kenyan rhizobia analyzed in this study (NAK103, NAK287, NAK227, NAK288 and NAK378) were more competitive at nodulating *P. vulgaris* than CIAT899 and these strains are also effective at fixing N_2_ with *P. vulgaris* (Mwenda et al. 2018). Thus, Kenyan soils do harbor competitive and effective rhizobia for this important grain legume. These five strains are therefore candidates for further inoculant development evaluations and may prove better adapted to the local environment of east Africa than CIAT899, which originates from Colombia (Martínez-Romero et al. 1991). Indeed, several studies have used a similar approach of identifying locally adapted inoculant strains for cultivation of *P. vulgaris* in Spain (Mulas et al. 2015; Pastor-Bueis et al. 2019). However, this study also found that two strains that were as competitive (NAK266 and NAK312), and three strains that were more competitive (NAK315, NAK332 and NAK368) than CIAT899, were only partially (NAK315, NAK266 and NAK312) or poorly (NAK332 and NAK368) effective at fixing N_2_. The presence of these suboptimal yet competitive strains in soils where *P. vulgaris* is cultivated could inhibit inoculation responses and result in diminished benefits from symbiotic N_2_ fixation. Future studies should therefore investigate the extent to which suboptimal and competitive rhizobia are present across areas of *P. vulgaris* cultivation in Kenya. If they prove widespread, the recent development of a reporter plasmid for high-throughput simultaneous measurement of rhizobial competitiveness and N_2_ fixation (Mendoza-Suarez et al. 2020), may well provide a valuable tool to identify elite inoculant strains for *P. vulgaris* in Kenya.

## Data Availability

All data generated or analysed during this study are included in this published article.
